# Electrical Double Percolation of Polybutadiene/Polyethylene Glycol Blends Loaded with Conducting Polymer Nanofibers

**DOI:** 10.3390/polym12112658

**Published:** 2020-11-11

**Authors:** Jun Morita, Takanori Goto, Shinji Kanehashi, Takeshi Shimomura

**Affiliations:** Graduate School of Engineering, Tokyo University of Agriculture and Technology, Koganei, Tokyo 184-8588, Japan; Jun_Morita@jsr.co.jp (J.M.); goto-tkn@awi.co.jp (T.G.); kanehasi@cc.tuat.ac.jp (S.K.)

**Keywords:** poly(3-hexylthiophene), nanofiber, composite film, double percolation, FET

## Abstract

The critical phenomena of double percolation on polybutadiene (PB)/polyethylene glycol (PEG) blends loaded with poly-3-hexylthiophene (P3HT) nanofibers is investigated. P3HT nanofibers are selectively localized in the PB phase of the PB/PEG blend, as observed by scanning force microscopy (SFM). Moreover, double percolation is observed, i.e., the percolation of the PB phase in PB/PEG blends and that of the P3HT nanofibers in the PB phase. The percolation threshold (*φ*_c_^I^) and critical exponent (*t*^I^) of the percolation of the PB phase in PB/PEG blends are estimated to be 0.57 and 1.3, respectively, indicating that the percolation exhibits two-dimensional properties. For the percolation of P3HT nanofibers in the PB phase, the percolation threshold (*φ*_c_^II^) and critical exponent (*t*^II^) are estimated to be 0.02 and 1.7, respectively. In this case, the percolation exhibits properties in between two and three dimensions. In addition, we investigated the dimensionality with respect to the carrier transport in the P3HT nanofiber network. From the temperature dependence of the field-effect mobility estimated by field-effect transistor (FET) measurements, the carrier transport was explained by a three-dimensional variable range hopping (VRH) model.

## 1. Introduction

Conductive polymer composites (CPC) that comprise immiscible polymer blends and conductive fillers are novel composite materials, featuring advantages of both polymer blends and conductive fillers. Generally, by loading conductive fillers such as carbon black (CB) [1,2,3,4,5,6,7,8], carbon nanotubes (CNTs) [9,10,11,12,13,14], and graphene [5], it was reported that the resultant mechanical strength and electrical properties can be drastically modified. In particular, the conductivity can be changed from that of an insulator to that of a conductor by increasing the amount of fillers. Furthermore, fillers have been reported to be located selectively in a specific phase [1,2,3,5,7,10,11,13,14] or at the interface of the macro-phase separations [1,4,6,8,9,12]. This selective localization of conductive fillers contributes to reducing the amount of fillers required for obtaining conductive properties.

Sumita et al. reported that CPCs with blended matrices have two different hierarchies of percolation at different scales, known as double percolation [1]. When loaded in high-density polyethylene (HDPE)/poly(methyl methacrylate) (PMMA) blends, CB was selectively located in the HDPE phase of macro-phase separation, and the HDPE phase was estimated to be the conductive phase. In this case, the electrical conduction was determined by the percolation of CB in the HDPE phase and the percolation of the conductive HDPE phase in HDPE/PMMA blends. In this system, the conductivity increased significantly at a low volume fraction of CB less than 0.05–0.1, which corresponds to the conductive percolation threshold *φ*_c_. In the double percolation system using CNTs as a filler, a *φ_c_* value lower than that of CB was expected owing to the small diameter and high aspect ratio of CNTs. Petra et al. reported an extremely smaller *φ*_c_ = 0.08 wt% of CNTs than that of CB in a polypropylene (PP)/HDPE blended matrix [10]. Yan et al. investigated the difference in *φ*_c_ between CNTs localized in a specific phase or at the interface of macro-phase separation using PMMA-modified CNTs in poly(vinylidene fluoride) (PVDF)/polystyrene (PS) blends [12]. The *φ*_c_ of the localization at the interface (0.07 wt%) was 50% lower than that in the PS phase (0.17 wt%). 

Although there have been various studies on double percolation, both *φ*_c_ and the critical exponent *t* of both percolations have not been comprehensively clarified. Furthermore, as inorganic fillers such as CB and CNT tend to easily aggregate in the polymer matrix, *t* of the percolation of fillers was frequently reported to be larger than the ideal value estimated from the percolation theory. Dai et al. reported that the *t* of CB in polyethylene terephthalate (PET)/polyethylene (PE) was 6.4, which was much larger than the ideal value of 2.0 for three-dimensional percolation [6]. For a comprehensive understanding of double percolation, conductive fillers with good affinity to the polymer matrix are required, which can disperse well in the matrix.

Conducting polymer nanofibers have attracted increasing interest owing to their unique shapes and electrical properties for various applications such as, in molecular wires, organic transistors, and sensors [15,16,17]. They are candidates for their use as fillers in CPCs. In particular, nanofibers of regioregular poly(3-hexylthiophene) (P3HT) crystallized from supercooled solutions in adequate solvents exhibit a fine whisker-like structure with a width of 15 nm and length of several µm [18]. These nanofibers can function as the p-type active layer of a field-effect transistor (FET) with a considerably high field-effect mobility, comparable to the best reported yet for P3HT films [19,20,21,22,23,24]. In addition, adequate doping could induce P3HT nanofibers with sufficient conductivity. Recently, it was reported that P3HT was recrystallized as nanofibers in conventional polymers such as PMMA and PS, resulting in nanofiber CPC [25,26,27,28,29,30,31,32]. Such nanofiber CPCs exhibit semiconducting properties and can be fabricated into a flexible FET by simply attaching electrodes [25,26,27,28,29]. An effective P3HT nanofiber network percolated into the bulk matrix of PMMA was observed using Kelvin probe force microscopy [29]. Furthermore, a similar nanofiber network of poly(3-butylthiophen) (P3BT) was observed using conductive atomic force microscopy [33]; this nanofiber CPC showed potential to be formed into flexible films with both high mechanical strength and high conductivity, with sufficient environmental stability [25,26,31]. 

In this study, we investigated polybutadiene (PB)/polyethylene glycol (PEG) blends loaded with P3HT nanofibers, to reveal the critical phenomena of the double percolation in all polymer systems that include fillers. We chose PB as a polymer with good affinity to P3HT, and PEG as a polymer with poor affinity to P3HT, both forming the blended matrix of CPC, featuring a macroscopic phase-separated structure. Although double percolation using P3HT nanofibers has not been investigated yet, the critical phenomena (*φ*_c_ and *t*), which are strongly related to the percolation dimensionality of double percolation, can be investigated in detail by comparison with the ideal value of percolation. P3HT nanofibers have good affinity with some polymers, and can be dispersed uniformly in the polymer matrix. Furthermore, the temperature dependence of the field-effect mobility of carriers reflects the dimensionality of the carrier transport. By using polymer blends loaded with P3HT nanofibers, we can investigate the multi-scale dimensions of carrier conduction.

## 2. Materials and Methods 

### 2.1. Preparation of PB/PEG Blends Loaded with P3HT Nanofibers

Regioregular P3HT (*M*_w_ 44,000), PB (*M*_w_ 200,000), and PEG (*M*_w_ 10,000) were purchased from Sigma-Aldrich Co. Inc. (St Louis, MO, USA) and used without further purification. Chloroform and anisole were purchased from FUJIFILM Wako Pure Chemical Co. Ltd. (Tokyo, Japan) and Kokusan Chemical Co. Ltd. (Tokyo, Japan), respectively. 

The affinity between the P3HT nanofiber and PB or PEG was determined by scanning force microscopy (SFM) of the P3HT/PB or P3HT/PEG composites, as shown in Figure S1 in Supporting Information A. In P3HT/PB, P3HT nanofibers were dispersed uniformly in the PB matrix, whereas in P3HT/PEG, nanofibers were aggregated and distributed unevenly in the PEG matrix. PB/PEG blends loaded with P3HT nanofibers were prepared in accordance with the procedure reported in the cases of polymethyl methacrylate (PMMA) or polystyrene (PS) as the matrix [25,26,29]. P3HT, PB, and PEG powders were added to a solvent mixture composed of chloroform, as a good solvent of P3HT, and anisole, as a poor solvent of P3HT, stirred at 60–70 °C for 60 min. The weight ratio of P3HT was fixed at 0.05 wt% in all samples, with 70:30 (*v*/*v*) solvent mixture of chloroform/anisole. For varying the blended matrix composition, we prepared solutions with a volume fraction of *φ*_PB_ = *V*_PB_/(*V*_PB_ + *V*_PEG_) of approximately 0.22–0.93 with a fixed ratio of P3HT to the matrix of 10 wt%. *V*_PB_, *V*_PEG_, and *V*_P3HT_ are the volume of PB, PEG, and P3HT components, respectively, estimated from the weight and density of each component. On the other hand, for varying the density of P3HT nanofibers in the blended matrix, we prepared solutions with a volume fraction of *φ*_P3HT_ = *V*_P3HT_/(*V*_PB_ + *V*_P3HT_) of 0.02–0.30 with a fixed matrix composition (*φ*_PB_ = 0.72–0.73). As P3HT had a good affinity with the PB component to be localized in the PB phase, we used the volume fraction *φ*_P3HT_ instead of *V*_P3HT_/(*V*_PB_ + *V*_PEG_ + *V*_P3HT_). Each solution was cooled gradually to 20 °C at a rate of 25 °C/h without stirring. Over one week of incubation, the transparent yellow solution turned into a turbid reddish-brown suspension, indicating fiber formation. Films of PB/PEG blends loaded with P3HT nanofibers were prepared by spin casting the suspensions at 2000 rpm for 90 s on a substrate, followed by removal of the residual solvent by vacuum drying.

### 2.2. Characterization of PB/PEG Blends Loaded with P3HT Nanofibers

Pt electrodes with a length of 0.6 mm and a gap of 40 µm were fabricated with a bottom-contact configuration by sputter deposition (EIS-200ER, Elionix Inc., Tokyo, Japan), using a shadow mask on a piece of doped Si wafer with a 255 nm SiO_2_ layer that was thermally grown on top (SiO_2_/Si), purchased from SEIREN KST Corp. (Fukui, Japan), as shown in Figure S2 in Supporting Information B. 

The microscopic structure of the composites was observed in air with a scanning probe microscope (SPM; Nanocute/NanoNavi IIe, Hitachi High-Tech Science Corp., Tokyo, Japan) under the SFM mode. The instrument was equipped with a commercial silicon cantilever (OMCL-AC160TS-C3, Olympus Corp., Tokyo, Japan) with a spring constant and a resonant frequency of approximately 26 N/m and 300 KHz, respectively. The thickness was measured by a stylus profilometer (Dektak XT-S, Bruker Japan Inc., Yokohama, Japan).

Field-effect transistor (FET) measurements were conducted by a two-probe method in vacuum below 10^−5^ Torr, using a system combining a cryogenic probing station (LIPS, Nagase Techno-Engineering Co. Ltd., Tokyo, Japan) and the Keithley model 236 source measure unit (SMU) (Keithley Instruments, Inc., Cleveland, OH, USA) to measure the source–drain characteristics, and a Keithley 2400 digital source meter (Keithley Instruments, Inc., Cleveland, OH, USA) for applying the gate voltage.

## 3. Results and Discussion

### 3.1. Selective Localization of P3HT Nanofibers in PB/PEG Blends

**Figure 1:**
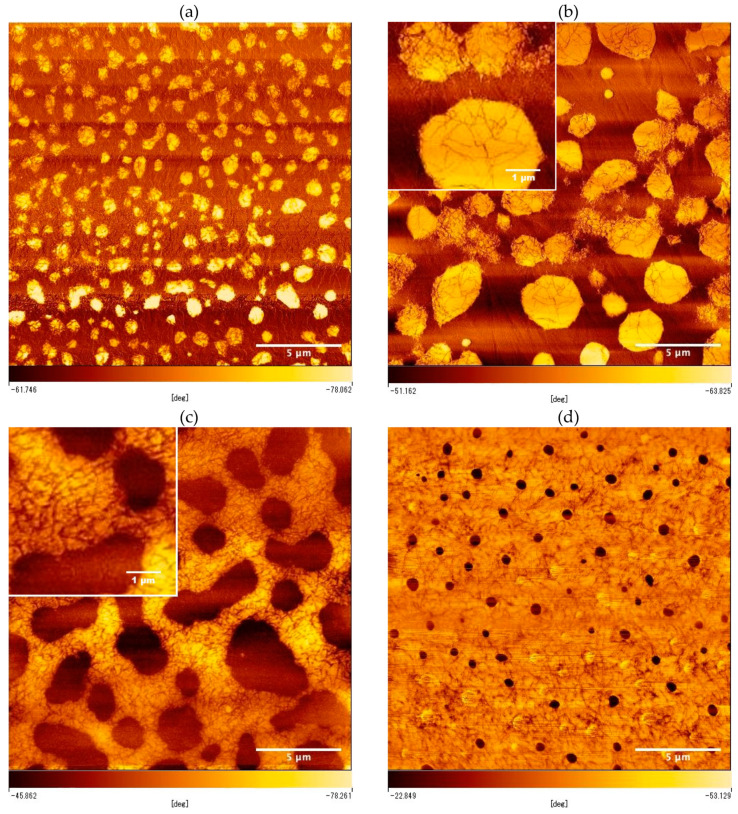
The SFM phase images of PB/PEG blend films with P3HT nanofiber, with *φ*_PB_ values of (**a**) 0.22, (**b**) 0.40, (**c**) 0.57, and (**d**) 0.72 (20 µm × 20 µm). Top left inset of (**b**,**c**) shows the magnified image of the phase image. From these images, P3HT nanofibers were confirmed to be selectively localized in the PB phase.

To confirm the distribution of P3HT nanofibers in the PB/PEG blended matrix, SFM observation of the thin-film of the composites was performed by changing the blend ratio of PB/PEG. Figure 1 shows SFM phase images with changing *φ*_PB_ from 0.22 to 0.72. Through all images, the sea-island structure originating from the macro-phase separation was observed. In all images, the bright phase corresponded to the PB component. For low values of *φ*_PB_ (Figure 1a,b), the PB component formed the island phase in the sea phase of the PEG component, whereas for high values of *φ*_PB_ (Figure 1c,d), the PB component formed a continuous sea phase. The crossover point of the island (minor) phase to the sea (major) phase of the PB component was at *φ*_c_ of between 0.40 and 0.57, and a continuous structure of the PB phase could be observed at *φ*_PB_ greater than *φ*_c_. Furthermore, it was found that P3HT nanofibers were positioned mostly in the PB component of the sea-island structure. P3HT nanofibers were mostly localized in the island phase at *φ*_PB_ < *φ*_c_, while nanofibers were mostly localized in the continuous sea phase at *φ*_PB_ > *φ*_c_, indicating that P3HT nanofibers were selectively partitioned into the PB phase. In particular, the percolation behavior of the PB phase in PB/PEG blends selectively embedded with P3HT nanofibers could be observed with increasing *φ*_PB_.

### 3.2. Electrical Conductivity of PB/PEG Blends Loaded with P3HT Nanofiber

**Figure 2:**
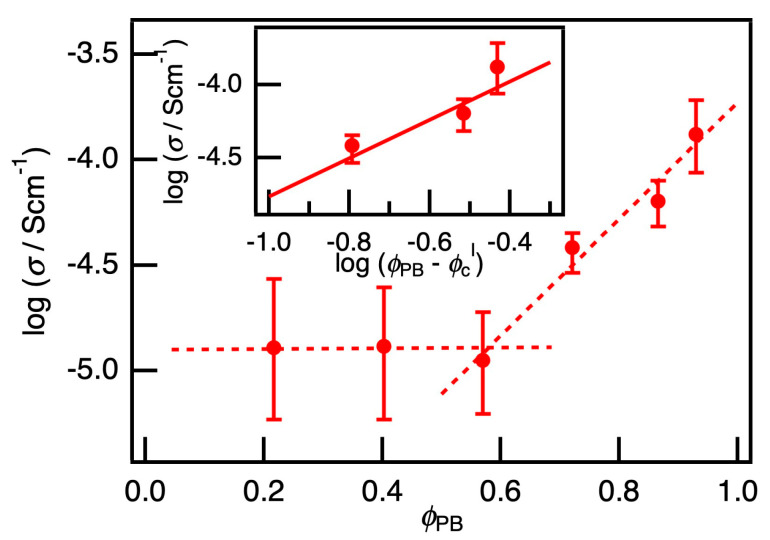
The volume fraction of PB component *φ*_PB_ dependence of the conductivity *σ*. The crossover point could be observed at the percolation threshold *φ*_c_^I^ = 0.57. The inserted chart shows log *σ* v.s. log (*φ*_PB_ – *φ*_c_^I^). From a slope, the critical exponent *t*^I^ can be estimated to be 1.3.

The electrical conductivity of PB/PEG blends loaded with P3HT nanofibers was investigated. With increasing *φ*_PB_ at a fixed P3HT ratio, the percolation of the PB phase in the PB/PEG blends were observed as an electrical percolation of the conductivity *σ* because the PB phase selectively embedded with P3HT nanofibers can be regarded as a conductive phase. Figure 2 shows the *φ*_PB_ dependence of *σ* of the PB/PEG blends loaded with P3HT. The PB/PEG blends had almost constant *σ* values in the low *φ*_PB_ region, while *σ* increased drastically with *φ*_PB_ in the region above the crossover point at *φ*_BR_ = 0.57. When *φ*_PB_ < 0.57, PB phase as a minor component formed isolated island, and the electrical conduction suggested to be dependent on the sea phase of PEG component, which embedded with P3HT, only to a small extent, connecting the conductive PB islands. As the PB phase has a continuous structure at *φ*_PB_ over 0.57, *σ* increases due to the percolation of the PB phase in the PB/PEG blends.

Here, *φ*_PB_ = 0.57 can be regarded as the percolation threshold *φ*_c_^I^. In the percolation model, *σ* follows the relation $\sigma \sim {\left(\phi_{\mathrm{PB}}-\phi_{c}^{I}\right)}^{t^{I}}$; thus, a critical exponent *t*^I^ could be estimated to be 1.3 from the slope of the inserted chart in Figure 2. In the percolation model, it is known that two-dimensional percolation theoretically has a critical exponent of 1.3 [34]; hence, this composite film evidently exhibits two-dimensional percolation of the PB phase. Here, the average diameter of the islands of the PB component in Figure 1b was estimated to be 4.4 µm, which corresponded to a characteristic length of the sea-island structure around the critical point. The characteristic length was much larger than the film thickness of approximately 60–100 nm, and the degree of freedom was suppressed in the direction of the thickness; therefore, it was evident that the percolation behaved as a two-dimensional system with *t*^I^ of 1.3. Furthermore, the percolation threshold *φ*_c_^I^ of 0.57 also indicated the two-dimensional percolation because *φ*_c_ of two- and three-dimensional percolation was theoretically estimated to be 0.45 and 0.16, respectively [35].

**Figure 3:**
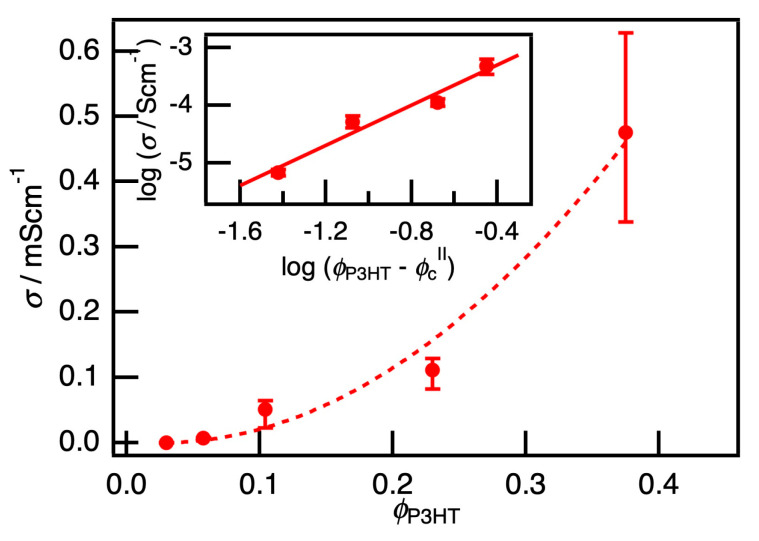
The volume fraction of P3HT nanofiber *φ*_P3HT_ dependence of the conductivity *σ*. The percolation threshold *φ*_c_^II^ was estimated to be 0.02. The inserted chart shows log *σ* v/s. log (*φ*_P3HT_ – *φ*_c_^II^). From a slope, the critical exponent *t*^II^ can be estimated to be 1.7.

On the other hand, increasing P3HT ratio in the matrix at a fixed PB/PEG ratio, drastically increased *σ*. Figure 3 shows the *φ*_P3HT_ dependence of the conductivity *σ* of the PB/PEG blends loaded with P3HT. *σ* was less than 10^−8^ S cm^−1^ in the region of *φ*_P3HT_ < 0.02. Therefore, the percolation threshold *φ*_c_^II^ was estimated to be 0.02, which was considerably smaller than *φ*_c_^I^. Generally, anisotropic fillers with small diameters and high aspect ratios, such as CNTs, are reported to exhibit a low percolation threshold from 0.08 to 3 wt% in CPC with a blended matrix [8,9,10,11,12,13,14]. Furthermore, in our previous studies on composite films of P3HT nanofibers in polymethacrylate (PMMA), a significant amount of conductivity was measured for the ratio of P3HT/PMMA at approximately 5 wt% [28]. The SFM observations at different ratios of P3HT/PMMA was reported previously [31]. The nanofiber network formation was observed above 1 wt% and nanofiber aggregation occurred above 10 wt%. The results of the present study show that the P3HT nanofiber with a diameter of approximately 15 nm [24] and an aspect ratio of more than 100 resulted in *φ*_c_^II^ = 0.02, which is consistent with the results of previous studies.

Conductivity *σ* is assumed to follow the relation $\sigma \sim {\left(\phi_{P3\mathrm{HT}}-\phi_{c}^{\mathrm{II}}\right)}^{t^{\mathrm{II}}}$, and the critical exponent *t*^II^ could be estimated to be 1.7 from the slope of the inserted chart in Figure 3. This value is between the theoretical critical exponent of two-dimensional percolation of 1.3 and three-dimensional percolation of 2.0 [36]; thus, these composites likely have percolation dimensionality that is between that of two and three dimensions. The length of the P3HT nanofiber was almost a few µm [24]. This length was comparable to the width of the continuous channel comprising the PB component approximately 2 µm, but was an order of magnitude larger than the film thickness. Thus, *t*^II^ of 1.7, which was larger than *t*^I^ and an intermediate value of two- and three-dimensional percolation, was agreeable. In previous studies of CPCs using CB or CNT as fillers, *t* was reported to be in the range of 1.3 to 6.4 [6,9,10,36]. Please note that a value of *t* much larger than 2.0 was frequently reported, which was far from the ideal value estimated by percolation theory, and was ascribed to the effect of aggregation on CB or CNT. In sharp contrast, our system presented in this work shows that the P3HT nanofibers were well-dispersed in the PB phase without apparent aggregation, resulting in a critical exponent within the ideal range.

**Figure 4:**
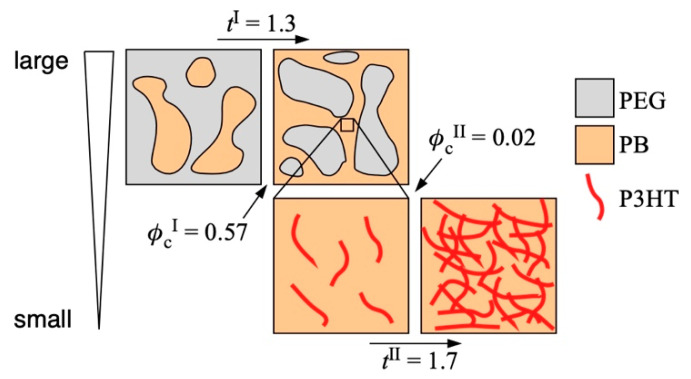
Schematic image of hierarchic percolations (double percolation) of the P3HT-nanofiber-filled PB/PEG blend composites.

The double percolation, percolation thresholds, and critical exponents of both percolations observed in our present system are summarized in Figure 4. The percolation of the PB phase loaded with the P3HT nanofiber in the PB/PEG blend was observed at a larger scale. *φ*_c_^I^ and *t*^I^ of this percolation were estimated to be 0.57 and 1.3, respectively, which corresponded to two-dimensional percolation. By contrast, the percolation of the P3HT nanofiber network in the PB phase was observed at a smaller scale. *φ*_c_^I^ and *t*^I^ of this percolation were estimated to be 0.02 and 1.7, respectively, which was regarded as the percolation with intermediate dimensionality between two and three. The values characterizing the critical phenomena of our system were in the theoretical estimation, unlike inorganic fillers such as CB and CNT. This ideal behavior was ascribed to the characteristics of our system being the composition of all polymer materials, including fillers well-dispersed in a selective phase.

### 3.3. Field-Effect Mobility of PB/PEG Blends Loaded with P3HT Nanofiber

**Figure 5:**
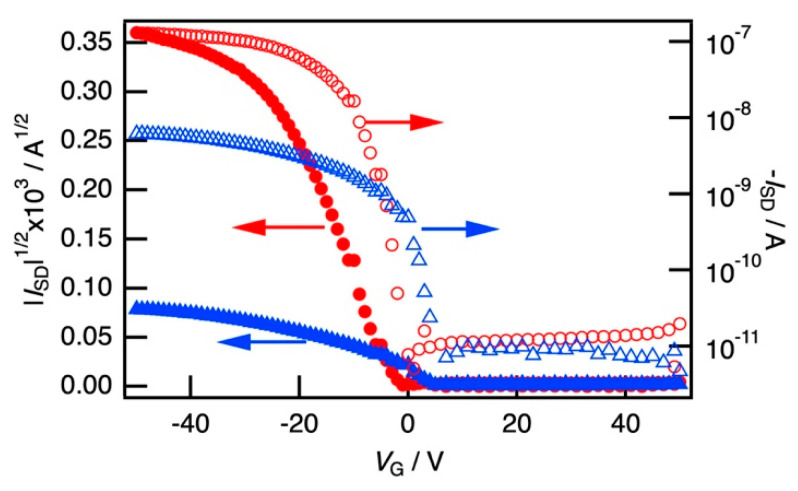
Transfer characteristics of the film of P3HT nanofiber composited in PB/PEG blend with *φ*_PB_ below (*φ*_PB_ = 0.40; filled and open triangle) and above (*φ*_PB_ = 0.72; filled and open circle) the critical threshold *φ*_c_^I^. In both cases, a typical p-type property was observed, showing marked amplification of *I*_DS_ with respect to the negative gate voltage *V*_G_. *μ* increased by an order of magnitude at *φ*_c_^I^.

To investigate the carrier transport around the critical threshold, FET measurements of the PB/PEG blend films loaded with P3HT nanofibers were performed. Figure 5 shows the transfer characteristics of the sample with a fixed P3HT ratio to the matrix (approximately 10 wt%) but a different *φ*_PB_ = 0.40 and 0.72, which are below and above the critical threshold *φ*_c_^I^, respectively. In both cases, a typical p-type property was observed, where the marked amplification of *I*_DS_ with respect to the negative gate voltage *V*_G_, but the on-off ratio of 30 and 13 was very small. Here, the field-effect mobility (*μ*) was determined using the following relation:

(1) $$\mu =\frac{2L}{WC_{\mathrm{OX}}}{\left(\frac{\partial \sqrt{I_{\mathrm{DS}}}}{\partial V_{G}}\right)}^{2},$$

where *L* is the spacing between the electrodes, *W* is the width of the electrodes, and *C*_ox_ is the capacitance of the insulation layer of SiO_2_ (255 nm thick), shown in Table S1 in Supporting Information B. From the transport characteristics, *μ* was estimated to be 1.85 × 10^−4^ cm^2^ V^−1^ s^−1^ (*φ*_PB_ = 0.40) and 2.86 × 10^−3^ cm^2^ V^−1^ s^−1^ (*φ*_PB_ = 0.72), indicating that *μ* increased by an order of magnitude at *φ*_c_^I^. In the region of *φ*_PB_ < *φ*_c_^I^, the conducting PB component formed a separated island phase and slightly embedded P3HT in the PEG sea phase connected with the PB islands. Carrier transport in the sea phase is ascribed to the rate-limiting step of conduction and decreases the carrier mobility. On the other hand, in the region of *φ*_PB_ > *φ*_c_^I^, *μ* was comparable to that of P3HT nanofibers composited in PMMA single matrix reported previously [25,26,27,28]; hence, the carrier conduction was scarcely affected by the PEG island phase.

**Figure 6:**
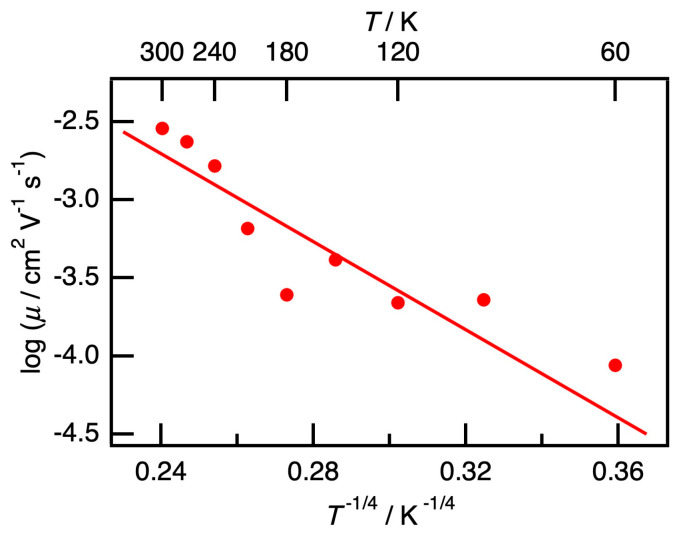
Temperature dependence of *μ* of P3HT nanofiber composited in PB/PEG blend with *φ*_PB_ = 0.72 (> *φ*_c_^I^). The dependence log *μ* against *T*^−1/4^ was almost linear one, thus the carrier transport was suggested to be three-dimensional variable range hopping (VRH) type.

The field-effect mobility of the PB/PEG blend film with *φ*_PB_ = 0.72 (>*φ*_c_^I^) loaded with P3HT nanofiber at a fixed P3HT to matrix ratio (approximately 10 wt%) was measured from 60 K to 300 K, and the results are shown in Figure 6. With increasing temperature, *μ* increased as thermo-activated behavior. The relationship of log *μ* with *T*^−1/4^ was almost linear; hence, the carrier transport was suggested to be a three-dimensional variable range hopping (VRH) type. This VRH relationship is consistent with that of various types of conducting polymers reported previously [37], and the effect of the matrix on the carrier conduction thus could not be observed. As the carrier transport can be regarded as a percolation of carriers in the nanofiber network, we could see three types of percolation with different scales in PB/PEG blends loaded with P3HT nanofibers, and the dimension decreased from three to two with an increase in the observation scale.

## 4. Conclusions

We investigated the critical phenomena of double percolation on polybutadiene (PB)/polyethylene glycol (PEG) blends loaded with poly-3-hexylthiophene (P3HT) nanofibers. P3HT nanofibers were selectively located in the PB phase of the PB/PEG blends, and hence the PB phase could be regarded as a conductive phase. In this system, double percolation phenomena could be observed. The percolation threshold of the PB phase in the PB/PEG blends was *φ*_c_^I^ = 0.57, and the critical exponent *t*^I^ was estimated to be 1.3. These critical values indicated two-dimensional percolation. The film thickness of approximately 60–100 nm, which was considerably smaller than the characteristic length of the PB islands, also indicated that the percolation behaved as a two-dimensional system. On the other hand, the percolation threshold of the P3HT nanofiber in the PB phase was *φ*_c_^II^ = 0.02, and the critical exponent *t*^II^ was estimated to be 1.7. As the length of the P3HT nanofiber was comparable to the width of the continuous channel comprising the PB component, it was an order of magnitude larger than the film thickness. This was in agreement with that *t*^II^ of 1.7 was an intermediate value between two- and three-dimensional percolation. As a result, the critical phenomena of double percolation on polymer blends loaded with P3HT nanofibers can be clearly explained by the percolation theory. In addition, the temperature dependence of the field-effect mobility estimated by FET measurement was also explained by the three-dimensional VRH model. As the carrier transport can be regarded as a percolation of carriers in the nanofiber network, we could see multi-percolation with different scales in PB/PEG blends loaded with P3HT nanofibers.

## Supplementary Materials

The following are available online at https://www.mdpi.com/2073-4360/12/11/2658/s1, Figure S1: The SFM phase images of P3HT nanofiber composited in (a) PB and (b) PEG matrix (20 µm × 20 µm). From the images, P3HT nanofibers were uniformly dispersed in PB matrix while nanofibers aggregated each other in PEG matrix., Figure S2: Optical microscope images of a substrate with electrodes for measuring conductivity and estimating the field-effect mobility, Figure S3: Temperature dependence of the transfer characteristics of the PB/PEG blend film loaded with P3HT nanofiber with φPB = 0.72 (>φcI) from 60 K to 300 K, Table S3: The spacing between the electrodes L, the width of the electrodes W, and the capacitance of the insulation layer of SiO_2_ (255-nm thick) Cox.
