# Supplementary material for: Electrical Double Percolation of Polybutadiene/Polyethylene Glycol Blends Loaded with Conducting Polymer Nanofibers
Source: Polymers (Basel). 2020 Nov 11;12(11):2658. doi: 10.3390/polym12112658 (PMC7696799; doi:10.3390/polym12112658)
Supplement: Supplementary file 1 [file polymers-12-02658-s001.pdf]

polymers

MDPI

Article

Supporting Information

Electrical Double Percolation of Polybutadiene/Polyethylene Glycol Blends Loaded with Conducting Polymer Nanofibers

Jun Morita [1], Takanori Goto [1], Shinji Kanehashi [1] and Takeshi Shimomura [1,*]

[1] Graduate School of Engineering, Tokyo University of Agriculture and Technology, Koganei, Tokyo 184-8588, Japan; Jun_Morita@jsr.co.jp (J.M.); goto-tkn@awi.co.jp (T.G.); kanehasi@cc.tuat.ac.jp (S.K.)

* Correspondence: simo@cc.tuat.ac.jp; Tel.: +81-42-388-7051

Received: 27 October 2020; Accepted: 10 November 2020; Published: date

A. SFM Image of P3HT Nanofiber Composited in a Single Component

To clarify the affinity between the P3HT nanofiber and PB or PEG, composites of P3HT/PB (= 0.1 w/w) and P3HT/PEG (= 0.1 w/w) were prepared, and surface observation was performed via scanning force microscopy (SFM). Since P3HT nanofibers were dispersed almost uniformly in the PB matrix, from the phase image of Figure S1a, P3HT was found to have a good affinity for PB. On the other hand, the P3HT nanofibers aggregated in the PEG matrix from the phase image of Figure S1b, indicating that P3HT had a poor affinity to PEG. From this result, it was expected that P3HT nanofibers were selectively localized in the PB phase of the macro-phase separation of PB/PEG blends.

(a) (b)

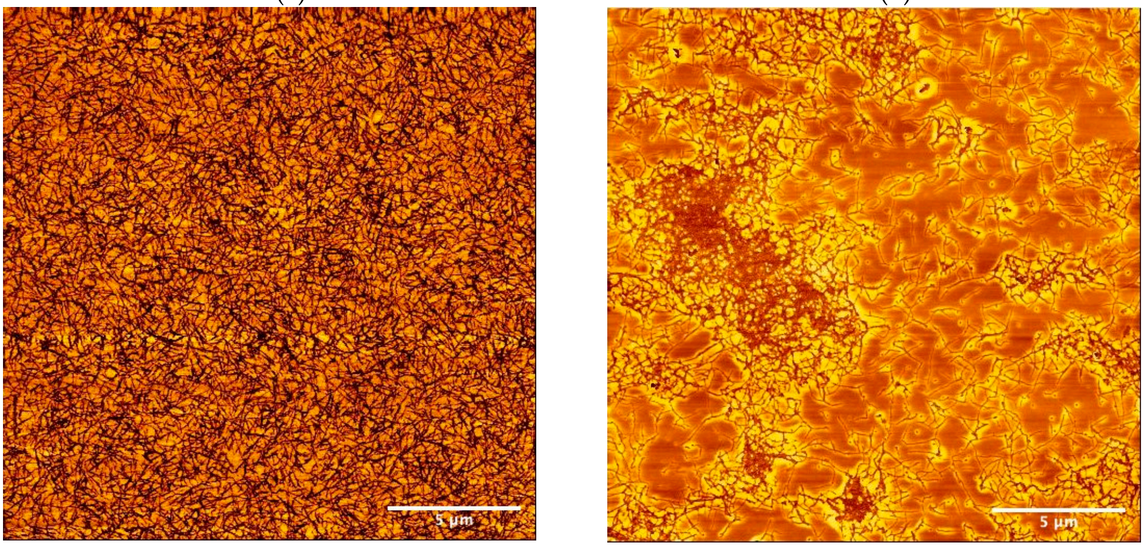

Figure S1. The SFM phase images of P3HT nanofiber composited in **a**) PB and **b**) PEG matrix (20 μm × 20 μm). From the images, P3HT nanofibers were uniformly dispersed in PB matrix while nanofibers aggregated each other in PEG matrix.

B. Parameters and Substrate with Electrodes for Estimating Field-Effect Mobility

The parameters for estimating the field-effect mobility are shown in Table S1, and optical microscope images of a substrate with electrodes for measuring conductivity and estimating the field-effect mobility are shown in Figure S2.

Table S1. The spacing between the electrodes *L*, the width of the electrodes *W*, and the capacitance of the insulation layer of SiO_2 (255-nm thick) C_{ox}.

L / µm	W / µm	C_{ox} / nFcm^{-2}
40	600	13.8

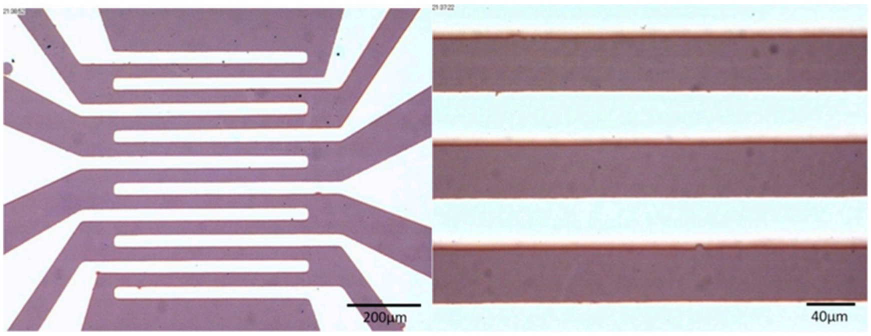

Figure S2. Optical microscope images of a substrate with electrodes for measuring conductivity and estimating the field-effect mobility.

C. Temperature Dependence of FET Property

For investigating the carrier transport in the PB/PEG blend film loaded with P3HT nanofiber, temperature dependence of FET characteristics was measured. The PB/PEG blend film with φ_{PB} = 0.72 (> φ_c^I) loaded with P3HT nanofiber at a fixed P3HT to matrix ratio (approximately 10 wt%) was prepared and the temperature dependence of the transfer characteristics was measured from 60 K to 300 K as shown in Figure S3. From this dependence, the temperature dependence of the field-effect mobility μ could be estimated.

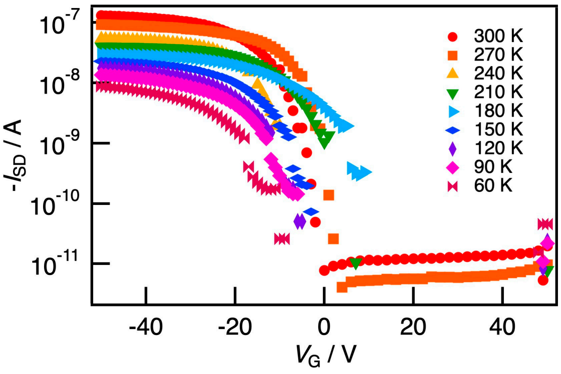

Figure S3. Temperature dependence of the transfer characteristics of the PB/PEG blend film loaded with P3HT nanofiber with φ_{PB} = 0.72 (> φ_c^I) from 60 K to 300 K.

© 2020 by the authors. Licensee MDPI, Basel, Switzerland. This article is an open access article distributed under the terms and conditions of the Creative Commons Attribution (CC BY) license (http://creativecommons.org/licenses/by/4.0/).
